# An Investigation of the Impact and Resilience of British High Streets Following the COVID-19 Lockdown Restrictions

**DOI:** 10.1007/s12061-022-09494-8

**Published:** 2022-11-29

**Authors:** Abigail Hill, James Cheshire

**Affiliations:** Department of Geography, University College London, London, England

**Keywords:** Retail resilience, High streets, Spatial analysis, Consumer data

## Abstract

British high streets have faced significant economic and cultural challenges as a consequence of the COVID-19 pandemic. This is predominantly due to government enforced restrictions which required all ‘non-essential’ retail to close, resulting in a significant change in the way consumers interacted with high streets. While all premises related to the retail or hospitality sector were forced to close, leading to rising vacancy rates, some high streets were more resilient to the economic shock than others. In this paper we detect some of the unforeseen consequences of the pandemic on British high streets and create a measure of resilience. The impact of the lockdown restrictions have resulted in some high streets, notably Spring Street in Paddington, London, experiencing disproportionate decline. Others including Northolt Road in Harrow, London were able maintain their occupancy. This study provides a typology of high street resilience incorporating the impact of the COVID-19 lockdown restrictions and links the impact of government policy to the economic performance of high streets. The outcomes from this research address both local and national policy contexts as the resilience typology has the potential to assist in funding allocation for recovery and regeneration projects.

## Introduction

Retail resilience is a notion that can be used to measure the persistence of retail areas to respond to economic or cultural shock (Holling, 1973; Hollnagel et al., 2006). While the term ‘resilience’ has a long presence in the fields of engineering and physical sciences, it was the 2008 recession that embedded the concept into the social sciences (Dawley et al., 2010). In particular, economic geographers have shifted their attention away from generating growth to ensuring resilience, resulting in debate around the term’s meaning and measurement (Dolega & Celińska-Janowicz, 2015). While there are many models for measuring resilience, each high street and its governing authority will have specific criteria to capture what is meant by associated terms such as sustainability and regeneration. That said, a national measure of resilience is a useful tool for planning since it enables a baseline for comparison between areas that can be used to benchmark successes and failures. It can also reveal the differences in high streets’ ability to withstand external shocks, which are often driven by the geographical contexts in which they are operating, a factor that can be overlooked in policy strategies (Whitworth, 2021).

The need for a national high street resilience measure has been catalysed the impacts of the UK Government’s restrictions enacted in response to the COVID-19 pandemic. On 24th March 2020 Britain went into the first COVID-19 lockdown, closing premises deemed ‘non-essential’ such as pubs, restaurants, gyms and other social venues. These were not fully free from restrictions to business practice until 12th April 2021 in England and Wales, when non-essential retail could reopen and 20th April 2021 in Scotland, when cafes, pubs and restaurants could reopen. The various lockdown restrictions across the three nations particularly impacted the leisure industry and also exacerbated high street competition with e-commerce as more people turned to delivery services. Meanwhile, stores offering ‘essential’ goods or services were allowed to remain open. Essential retailers included food shops, supermarkets, hardware stores, off-licences, petrol stations, vehicle repair services, banks, medical services, pet shops, launderettes, and funeral directors. In addition, pubs, bars and restaurants were allowed to remain open to offer takeaway food and drink in sealed containers (BBC, 2021). The COVID-19 lockdown restrictions also mandated ‘working from home’ unless workers met strict exemption criteria. Consequently, smaller towns and residential areas retained a population of commuters whose disposable income had previously led to predominant capital flows outside of local high streets (Crols & Malleson, 2018).

In order to fully assess the impact of lockdowns on British high streets, this research explores whether the COVID-19 pandemic compounded pre-existing inequalities for some areas while creating opportunity for others. In order to achieve this, we first develop a series of heuristics to measure high street resilience which include vacancy rates, occupier change and proportion of ‘essential’ stores. Secondly, the impact of specific categories of stores that were allowed to remain open in Britain during the restrictions and their impact on high street performance will be analysed. Finally, the study uncovers those high streets in Britain that were more adaptable to change during the COVID-19 pandemic.

## Literature Review

### The British Retail System

High streets have traditionally been a structural subsection of town or city centres in Britain, acting as focal points for work, leisure, shopping and housing (Phillips et al., 2021). Previous studies (Stocchi et al., 2016) have suggested that what consumers desire from British high streets is a holistic experience offering a mix of products, stores, and social experiences. Consumers also see value in high streets that can create novel and exciting experiences, alongside functional features such as efficient transportation systems and parking. More specifically, Theodoridis et al. (2017) found that high streets and town centres can have a vast array of desired functions for the local population including a supply of “wellbeing” related facilities, free activities for children, and attractions for younger people. Different types of retail centres have been found to react differently to changing consumer behaviours, therefore classification systems that draw distinctions between different types of centres are important (Dolega et al., 2016).

In the British context, the work of Reynolds and Schiller (1992) illustrates that at the start of the 1990s retail hierarchies could be entirely built upon town centres. Their research proposed a five-tier system comprising 370 minor ‘district centres’ and six ‘provincial cities’ determined by the number of retailers in each location. More than two decades later, Dolega et al.’s (2016) retail hierarchy was derived using a composite index based on centre size, a diversity index, proportion of leisure and anchor stores, and vacancy rates. However, Jones and Livingstone’s (2018) study criticised the more complex index for not considering the long-term changes to the variety and roles of shopping centres, as it is predominantly focused on town centres. A similar challenge faces this piece of research due to its emphasis on high streets.

Other hierarchies that are based on town centres as opposed to high streets include Mumford et al.’s (2021) classification of retail centres based on footfall signatures and volumes as a measure of attractiveness. However, footfall signatures do not necessarily directly reflect success, acting as more of an indicative measure with research often relying on qualitative data from local stakeholders to validate models (Crols & Malleson, 2019). Dolega et al. (2021) have also utilised new forms of data to classify shopping and consumption spaces across Britain. The study develops a dynamic taxonomy in the form of a two-tier classification with 5 distinct clusters and 15 additional nested sub-clusters. While the study determines key characteristics relating to each retail area such as vacancy rate, type of retail and diversity, the typology is not directly related to the COVID-19 restrictions. Consequently, a retail hierarchy recognising the specific stores allowed to remain open between the time span of retail and leisure restrictions - March 2020 to May 2021 - is needed in order to capture the structural changes caused by the COVID-19 pandemic.

### High Street Resilience

Within the wider literature and government policy reports (Wiley & Lambiri, 2014; GOV.UK, 2021) and consequently within this paper the terms ‘high streets’ and ‘town centres’ are used in conjunction with each other. The rationale behind the merged or interchanging definition is because while some ‘secondary centres’ are not locally considered as main town centre high streets, they can often be more robust to economic crisis and change (Findlay & Sparks, 2012). Consequently, a town centre or high street’s resilience can be defined as the ability of the area to return to pre-shock conditions or to adapt into a new environment brought about by wider social and economic change (Fernandes & Chamusca, 2014; Hudson, 2010). The 2008 recession exposed the socio and spatial inequalities across Britain and led certain high streets to have sharper rises in store vacancy, leaving policy makers to rethink visions for regeneration (Dolega & Celińska-Janowicz, 2015). Therefore, high street research aiming to encapsulate a wider understanding of resilience should include the area’s ability to sustain long-term development and adapt to its local population’s needs.

Studies specifically measuring the resilience of retail to lockdown restrictions include Appel and Hardaker’s (2021) qualitative analysis of the experiences of retailers in Germany through in-depth interviews. The study separates retailers’ resilience strategies into two categories – those who intend to return to their former stage of success utilising their pre-pandemic methods, and those who view the pandemic as an opportunity for fundamental change, such as pursuing an online approach in search of sustainable growth. The findings suggest that retail resilience research should consider two different aspects of resilience – firstly, the rate at which a high street can return to its original performance, and secondly, the ability of a high street to transform and adapt in line with wider social and cultural changes (Fernandes & Chamusca, 2014; Hudson, 2010). Dolega & Celińska-Janowicz (2015) have described a town centre’s period of relative stability as when there is low retail churn, high capacity and slow responsiveness to change. In contrast, ‘adaptive resilience’ is the state of continual adaption and redesign in pursuit of a core goal (Robinson, 2010). Consequently, local councils’ understanding of retail resilience is important; while there are many resilience models, each high street will have specific goals relating to the spectrum of self-preservation and redesign.

In the context of the COVID-19 pandemic and the restrictions that resulted from it, it is important to select appropriate performance measures that can distinguish the differing levels of resilience exhibited by individual high streets. The first crucial factor linked to high street success and resilience is the dominant store category. For example, Powe (2006) found that rural high streets with high individuality, cultural activities and eateries such as Hexham, Alnwick and Morpeth in Northumberland were found to attract a high volume of urban visitors. In addition, Dolega and Lord (2020) have found that retail composition is shaped through the growth of different retail types occurring at different rates, with different areas plateauing and expanding simultaneously in the same high street.

Generally, across most high streets there has been a reorientation towards leisure and convenience services driving positive growth in both sectors (Coca-Stefaniak, 2013). Consequently, Pinkerton et al.’s (1995) work on residents’ consumption behaviour within their local community could be applied to the present rise in convenience shopping. Pinkerton et al. (1995:1) suggest that consumers’ socio-economic status, personal location and community satisfaction influences whether an individual shops locally (‘inshopping’), and the range of goods and services available dictates (‘outshopping’). Consequently, a precise assessment of resilience could be made through building an understanding of the specific high street stores, including essential and convenience stores, that are available to the local population. By understanding existing retail structures and users, high street re-imagination strategies can be tailored to satisfy existing consumers or attract new ones (de Noronha et al., 2017).

The second important component of high street resilience is the vacancy rates of high streets before the COVID-19 pandemic. Prior to the pandemic, high streets had already been facing numerous problems, including loss of attractiveness to investors (Jones, 2010). Grenadier’s (1995) work on real estate cycles can be applied to explain the reluctance of landlords to fill vacant spaces in downward markets, as seen during the 2008 recession. This is because it is a more cost-effective option for landlords to retain the current environment of vacancy being enough to offset the possible benefits of filling a vacant space. Therefore, if high streets were already struggling with high vacancy rates, the economic shock caused by the pandemic may have exacerbated their inability to fill vacant premises. For example, following the 2008 recession, vacancy rates rose substantially, further intensified by multiple large national retailers falling into insolvency (Jones, 2010). The Local Data Company (LDC, 2009) reported that during the year after the recession hit, 10.8 per cent of high streets across the UK consisted of empty floor space. Nevertheless, LDC also reported that some towns displayed double the national average, revealing clear disparities in the impacts of the downturn.

Therefore, it is important to investigate high street vacancy rates before the pandemic and the degree to which they rose or fell following the easing of restrictions on non-essential retail in May 2021. The 2008 recession resulted in clothing, footwear and personal goods stores being hardest hit, while food and household stores had the lowest levels of closures (Thomas & Shah, 2009). Post-lockdown conditions may result in rises in vacancy due to similar patterns of store closures, especially since clothing and leisure facilities were among those forced to close.

One final indicator for high street resilience is occupier turnover. Such a measure is critical to understanding an area’s pace of change, controlling for other aspects including assets or unavoidable losses. A measure of occupier change should be used in conjunction with vacancy rates to measure and rank the speed at which retail locations change the business that occupies their premises. An exploration of high street resilience should be able to incorporate the probability of a shop thriving in an area and the consequential evaluation of consumer needs.

### COVID-19 Policy Impacts

Even before the COVID-19 lockdown restrictions triggered the local purchasing of essential items, smaller formats of grocery and convenience stores had been growing at a more rapid rate than large out-of-town supermarkets (Nielsen, 2017). Consequently, in 2016 IGD estimated that convenience stores accounted for 20.9% of food sales in the UK and predicted an 11.7% increase by 2021 (Wood, 2017). Furthermore, Dolega and Lord’s (2020) research also pointed to a rise in convenience culture and suggested that some retail areas that are in decline stabilise through a change in their wider retail environment that emphasises convenience stores, charity shops or discount stores.

In light of the COVID-19 lockdown restrictions leisure destinations in dynamic central settings suffered the largest reductions in activity, while retail centres which catered for the local population’s needs had a faster recovery in activity (Trasberg & Cheshire, 2021). Batty et al.’s (2021) analysis of Google Mobility Reports for London boroughs revealed that local grocery shopping declined the least out of all activity types during the lockdown to no less than 50% of normal activity. The analysis notably uncovers trends within the City of London, which experienced a pronounced drop to about 90% below normal activity before only returning to no more than 50% of the baseline, between 10th February 2020 up until the time of data collection on 18th October 2020.

Modified mobility patterns due to the lockdown restrictions have resulted in some areas recovering at faster rates than others. Quinio’s (2021) preliminary study found that Britain’s largest cities were the slowest to recover after the lockdowns. However, whilst Quinio suggests that large cities such as London, Manchester and Birmingham, or cities with many food and drink amenities, only require a return in footfall to recover, it may be undesirable for some smaller towns to return to their pre-pandemic state if they struggled with high vacancy and high occupier turnover.

While Quinio’s (2021) study utilises activity levels as a measure of resilience; there may be room to expand the analysis into a more concrete reflection of high street success. Other early studies into the impact of the pandemic, such as Gathergood et al.’s (2021) paper on the lockdown’s impact on consumer behaviour, begin to uncover the uneven regional nature of retail recovery. The strongest recovery has been in online spending in outer London as working from home practices have been seen to displace the location of spending. The study draws distinctions between online and offline spending during the second half of 2020. However, there is scope to explore whether there is a relationship between the retail composition of high streets and their associated resilience under lockdown.

This research aims to acknowledge and integrate current policy, which has adapted and responded to the changes created by the pandemic. One particular government response introduced in August 2020 to target the hospitality sector, which was particularly hard-hit by lockdown restrictions, was the Eat Out to Help Out scheme (GOV.UK, 2020). Government subsidies enabled participating businesses in Britain to offer a 50% discount from Monday to Wednesday with up to £10 off per person for food or non-alcoholic drinks consumed at the venue. González-Pampillón et al.’s (2021) study on the impact of the Eat Out to Help out scheme found that there was higher footfall associated with recreational activities on the days when the discount was available. However, the scheme was not a resounding success in terms of high street regeneration, with the study finding that on the whole it did not persuade people to use high streets for other purposes or to continue eating out once the subsidy had come to an end.

During the pandemic, city planning orientated around convenience also gained more traction. The “15-Minute City” originally proposed by Carlos Moreno in 2016 has increased in popularity due to its emphasis on proximity-centric planning, whereby neighbourhoods are planned to provide residents with basic essential services within a 15-minute walk or bicycle ride. The original concept has been reimagined and adapted to promote sustainability and resilience, and to emphasise identity in the post-pandemic era of town centres (Moreno et al., 2021). In particular, the authors argue that proximity-based planning may be more beneficial than aspects of the Smart Cities concept, which can arguably strengthen underlying social inequalities (Allam & Dhunny, 2019). Examples of local high street action adopted in response to the pandemic include the Mayor of London’s ‘High Streets for All Challenge’ that funded 35 projects, aiming to address local challenges and promote strategies to re-imagine high streets across London (Mayor of London, 2021). The Greater London Authority’s mission acknowledges that the pandemic-related challenges faced by high streets are uneven across the London boroughs. Such initiatives may therefore benefit from a quantitative measure of resilience which can be used to prioritise funding for individual high streets within boroughs that have been particularly impacted by the pandemic.

## Data and Methods

The data used for this research is provided by the Local Data Company (LDC) and comprises the location, occupier status (including if vacant) and retail category (e.g. pub, clothing store, restaurant) for 500,000 premises in Britain. This allows for the identification of stores that were allowed to remain open during the periods of lockdown covered in this study. The end date for the dataset is 28th June 2021.

In addition to the store location data, the Consumer Data Research Centre (2021) retail centre boundaries and classification system has been used to define the geographic extent of each of the high streets. The Consumer Data Research Centre (CDRC) retail boundaries were developed using open-source geo-coded retail unit location and land use data. The retail data was then aggregated to Uber’s (2018) Hexagonal Hierarchical Spatial Index (H3), which offers nested hexagon geometries at a range of granularities. The CDRC’s resulting hierarchical classification system is based on retail count, density and ranking within the respective local area to identify the prominence of each retail centre, aiming to capture variation between regional centres, market towns, small local centres, shopping centres, and retail outlets. This research selected the CDRC boundaries classified as either a district centre, local centre, major town centre, regional centre or town centre.

The five different categories have been included in order to encapsulate both ‘town centres’ and ‘secondary level’ high streets and local town centres within conurbations, local parades and neighbourhood centres. Other retail structures such as out of town retail parks are excluded (Wrigley & Lambiri, 2014:5). This is particularly important since secondary level high streets and local town centres are often under-reported and can be more resilient to economic shock (Findlay & Sparks, 2012).

The LDC retail location dataset was then joined with the selected CDRC retail boundaries. Only the boundaries that included 15 or more retail location points were used within the analysis, a threshold in line with the Office for National Statistics (ONS) and Ordnance Survey (OS) joint definition of a ‘high-street’ (Ordnance Survey, 2019). This resulted in 486 retail areas for use in this study.

The LDC store location data within each high street boundary was used to create 4 variables, summarised in Table 1: Occupier Change 2019, Vacancy 2019, Vacancy 2021 and Proportion of Essential Stores/ Services 2019. Firstly, to secure a robust basis for encapsulating the pre-pandemic occupier change, the first six months of 2019 have been selected as the baseline time period, due to January-June being the period in which most new businesses open for the first time (Aldermore, 2017). For January-June 2019 the number of new shops that opened was divided by the number of stores within the high street, to gain a measure of the percentage of premises which had new occupiers.

Secondly, for the same period of time, the number of shops that were vacant at the end of each month were divided by the number of stores within each high street, to gain a measure of the proportion of stores which were vacant. A six-month average for the vacancy of each high street was then calculated for January-June 2019.

Thirdly, to measure post-lockdown vacancy, the number of vacant stores at the end of June 2021 was divided by the number of stores within each high street. Due to inconsistent data collection during the lockdown and temporary closures of stores, June 2021 was used to calculate the post-lockdown measurement of vacancy. By the end of June 2021 all retail premises which had temporarily closed due to the lockdown restrictions would have re-opened by 17th May 2021.

While the data includes high streets in Wales and Scotland, where lockdown restrictions differed slightly during the pandemic, the distinctions between shops that were allowed to remain open between March 2020 and May 2021 were consistent. With this in mind the final attribute was calculated as the 6 month average (January- June, 2019) of the number of stores allowed to remain open during the various retail specific lockdown restrictions occurring between March 2020 and May 2021. The essential stores and services were divided by the number of stores within each high street. The ‘essential’ stores and services include cafés and coffee shops, as many remained open during lockdown for takeaways. Each of the store subcategories in the LDC data were manually added into a linear regression with high street-level vacancy from 2019. Each subcategory was then assessed as to whether it added to the fit of the model and increased the R2 value. The final subcategories deemed as ‘essential’ stores are displayed in full in Appendix 1.

**Table 1:** Variable specifications

Variable label	Attribute
Vacancy (2019)	6 month average for January- June, 2019 for number of vacant properties/ total stores within a high street.
Vacancy (2021)	Number of vacant properties/ total stores within high street for June 2021.
Occupier change (2019)	Number of new stores opened between January-June 2019/ total number of stores within a high street.
Essential Retail/ Services (2019)	6 month average for January- June, 2019 for number of stores allowed to remain open during lockdown restrictions/ total number of stores within a high street.

Assessing the impact of retail lockdown restrictions between March 2020 and May 2021 in Britain presents numerous empirical challenges, given that a number of factors may explain vacancy rates before the pandemic and additionally changes to vacancy following the lockdowns. Therefore, this research utilises the 4 high street characteristic variables to investigate the relationship between pre-pandemic vacancy, occupier change and ‘essential’ store and services proportion on post-restriction vacancy through using two ordinary least squares (OLS) regression models. The first model tests the hypothesis that pre-pandemic levels of occupier change, vacancy and essential retail proportions are associated with vacancy levels following the easing of lockdown restrictions. Consequently, the second model tests the hypothesis that pre-pandemic vacancy moderates the association between proportion of essential retail stores and services with post-restriction vacancy.

**Fig. 1:**
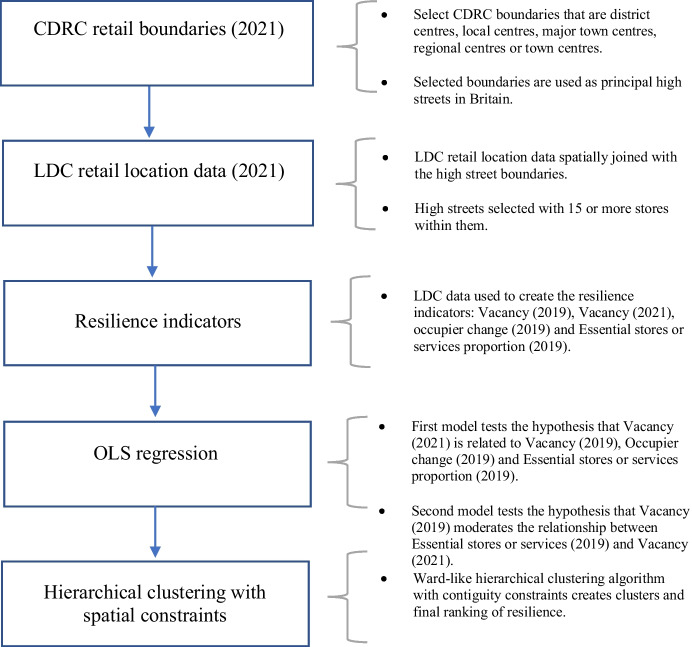
Overview of methodological process

Central to this study is the idea that we should account for the geographical distribution of performance-related characteristics. Literature relating to retail distribution and hierarchy has been drawn upon to select appropriate methods for the creation of a high street hierarchy. The majority of retail distribution models combine 20th Century land use theories with central place theory or growth pole theory (Forbes, 1972; Parr, 2017). Such broad and early analyses are conditional on simple datasets and consist of small snapshots in time, making it difficult to monitor the changing nature of the retail areas. Consequently, the final outcome of this research is the formation of a high street typology, using the LDC’s wide coverage and granular data and created using hierarchal clustering with spatial constraints. The ClustGeo package has been used for the implementation of a ward-like hierarchical clustering algorithm with soft contiguity constraints (Chavent et al., 2018). The package aims to increase spatial contiguity without deteriorating the quality of the homogeneity of the socio-economic variables included in the calculation. The ward-like hierarchical clustering approach has been selected to ensure that store locations with similar characteristics will not be placed into different clusters if they are spatially distant. Additionally, the method avoids the issue of having to define weights for geographical dissimilarities (Fig. 1).

## Descriptive Analysis

The granularity of the LDC data enables a straightforward distinction between stores forced to close and those essential stores allowed to remain open during lockdown. Descriptive statistics for all the variables can be found in Table 2. The overall vacancy rate for all high streets in 2019 was 9.0%, rising to 12.0% in 2021. The mean occupier change was 0.68% and the average essential retail proportion for British high streets was 43.61%.

**Table 2:** Descriptive statistics for the dependent and predictor variables

Variable	Average	Median	Standard deviation	Maximum	Minimum
Vacancy (2019) (%)	9.00	8.27	4.75	30.23	0.81
Vacancy (2021) (%)	11.95	10.62	5.82	34.20	1.55
Occupier change (2019) (%)	0.68	0.34	0.86	5.36	0.00
Essential retail and service proportion (2019) (%)	43.61	44.10	9.26	72.17	17.86

The Pearson’s correlation coefficients between the dependent variable, vacancy (2021) and the predictor variables of vacancy (2019), occupier change (2019) and essential retail and service proportion (2019) have been calculated and presented in Table 3. Vacancy in 2019 and percentage of essential stores and services within a high street were found to be significantly correlated to vacancy in 2021. The correlation coefficient between vacancy 2019 and vacancy 2021 is positive and indicates high temporal autocorrelation.

The correlation coefficient between essential retail and services % in 2019 and vacancy 2021 is negative. Meanwhile, occupier change, 2019 was found to be positively correlated with vacancy in 2019. Variance inflation factor (VIF) was used to test for multicollinearity, due to one of the correlation coefficients being as high as 0.8 (Menard, 2002).

**Table 3:** Pearson’s correlation coefficients between the dependent and predictor variables

	Vacancy 2021	Vacancy 2019	Occupier change 2019
Vacancy 2021			
Vacancy 2019	0.79**		
Occupier change 2019	0.06	0.13**	
Essential stores and services 2019	-0.56**	-0.40**	-0.08

Note: *Sig. < 0.05 **Sig. < 0.01 (two-tailed)

## Results

OLS regression has been conducted to investigate the influence of the 3 independent variables on vacancy in 2021. The results of estimating the stepwise OLS regression models are reported in Table 4. Model 1 shows the results for the direct effects of Vacancy %, Essential Retail/ Service % and Occupier Change % in 2019 on Vacancy 2021. It indicates a clear relationship between the predictor variables and the high street vacancy levels following the lockdown restrictions, despite other additional exogenous variables being excluded (e.g. local lockdown restrictions and local authority interventions). From Model 1 it can be observed that Vacancy 2019 has a strong significant effect on Vacancy 2021 (b = 0.84, p = < 2e-16). Additionally, % of essential stores and services in 2019 was found to have a moderate negative effect on Vacancy in 2021 (b=-0.18, p = < 2e-16), holding constant all other independent variables. Occupier change % was also found to have a moderate negative effect on Vacancy 2021 (b=-0.39, p = 0.023). For the independent variables in model 1, the highest VIF value of 1.21 was well below the cut-off measure of 10, suggesting that multicollinearity was not having a biasing effect on the OLS estimations (Menard, 2002).

Model 2 shows the results for the interaction effect between Vacancy and Essential Retail/ Service % in 2019. It can be observed that Vacancy 2019 retains its significant effect on Vacancy in 2021. In addition, the model shows that the interaction term is negatively and significantly associated with Vacancy 2021, therefore the strength of the association between Vacancy 2019 and Vacancy 2021 is weaker in high streets that had a higher proportion of stores that were allowed to remain open during the retail related lockdown restrictions.

One potential issue that arises within the two models is the use of the temporally lagged dependent variable (Vacancy 2021) with Vacancy 2019 as a predictor. Some research argues that the inclusion of a lagged dependent variable in regression models results in negatively biased coefficient estimates (Achen, 2000). However, more recent studies have noted that lagged dependent variables do not “dominate a regression” or make “real” effects disappear (Beck & Katz, 2011:350).

**Table 4:** British high street level vacancy for 2021 Estimation method: Robust OLS

Predictor variables	Model 1: Direct effects			Model 2: Interaction effect	
	Estimate	*p*-value	VIF	Estimate	*p*-value
Intercept	12.52***	< 2e-16		7.26***	9.17e-07
Vacancy (2019)	0.84***	< 2e-16	1.21	1.46***	< 2e-16
Essential stores and services (2019)	-0.18***	< 2e-16	1.19	-0.049	0.1338
Occupier change (2019)	-0.39*	0.023	1.02	-0.30	0.0742
Vacancy (2019) X Essential stores and services (2019)				-0.016***	5.47e-06
*n*	486			486	
R2	0.701			0.7136	
Adjusted R2	0.6991			0.7112	
P value	< 2.2e-16			< 2.2e-16	

*** 0.001, ** 0.01, *0.05 degree of significance

The second stage of this research was to create a geographical typology of high street resilience using all the indicators of resilience explored so far. The variables used within the hierarchical clustering with spatial constraints are Occupier Change, Essential Retail/Service % (2019) and Vacancy (2019). A typology has been devised based on pre-pandemic high street characteristics in order to evaluate which geographical areas of specific attributes had the lowest rise in vacancy. The method for conducting hierarchical clustering with spatial constraints outlined in Chavent et al. (2018) considers two dissimilarity matrices. Firstly, D0 is the Euclidean distance matrix between the *n* high streets determined by the variables, occupier turnover vacancy, and essential retail and services %. Secondly, D1 is the dissimilarity matrix used to account for the straight line Euclidean distances between the *n* high street centroid locations. D0 is equal to the occupier turnover and vacancy and essential retail and services distances and D1 is equal to the geographic distances between the municipalities. In order to select a suitable number of K clusters, a dendrogram for the high streets was created using D0 only. The dendrogram captures each hierarchical step in the model, allowing for different clustering solutions to be visually explored. Based on Kirk’s (2021) interpretation guidance the dendrogram favoured a four-cluster solution.

The introduction of a geographical constraint (D1) required a mixing parameter to set the importance of the constraint in the clustering process and improve the geographical cohesion of the four clusters, without jeopardising the cohesion between the vacancy, occupier change and essential retail and service percentages of the high streets. The geographic constraint is an important part of the resilience measurement, since it can be a proxy for for factors such as ‘linked shopping behaviour’, which is influenced by the proximity of high streets to larger retail centres (Wrigley et al., 2015). Additionally, research has found that while the resilience of individual retailers can be inadequate in responding to external shock, retailers even with an indirect association with the wider town centre or regional centre can benefit from what the network has to offer such as shared knowledge and partnerships (Edström, 2018). An *α* value of between 0 and 1 could be used to set the relative importance of the geographical constraints against the resilience variables whereby *α* = 0 meant no geographical constraints whilst *α* = 1 excluded the resilience variables. The separate calculations for the performance indicator homogeneity and for the geographic cohesion of the partitions obtained a range of values between 0 and 1 for the four high street clusters. The values were plotted and used to choose an *α* value that provides a compromise between the loss of performance indicator homogeneity and a gain of geographic cohesion. A value must be chosen that does not lose too much performance indicator value homogeneity but also increases geographic homogeneity. The value of *α* = 0.4 was chosen on this basis, resulting in a loss of homogeneity between the resilience indicator values of 22% but an increase of geographical homogeneity of 37%. New partitions were then obtained with *α* = 0.4 using the ‘hclustgeo’ function in the Clustgeo package to gain geographic cohesion.

Table 5 records the descriptive statistics for each hierarchical cluster formed with spatial constraints, alongside the geographic regions within Britain that the high streets are situated.

**Table 5:** Descriptive statistics for each of the high street clusters formed through hierarchical clustering with spatial constraints

Cluster	Regions		Occupier change (%)	Essential retail and services (%)	Vacancy (2019) (%)	Vacancy (2021) (%)	Vacancy Change (2019-21)
1	Yorkshire and the Humber, Wales, West Midlands, East Midlands, North West, South West, East of England, London, South East	Average Median Std. dev. Max. Min.	0.59 0.00 0.85 3.91 0.00	44.97 45.30 5.15 64.23 30.43	7.62 7.20 3.44 20.00 1.22	10.44 9.95 4.20 28.12 1.89	2.82 2.79 3.5 20.19 -8.49
2	Scotland, North West, North East, East Midlands, Yorkshire and the Humber, West Midlands	Average Median Std. dev. Max. Min.	0.83 0.67 0.86 5.36 0.00	36.29 35.35 6.20 51.94 25.56	13.88 13.87 5.66 30.23 1.37	17.76 18.49 6.79 34.2 5.33	3.88 4.06 3.10 9.92 -4.15
3	Wales, London, South East, South West, East of England, West Midlands	Average Median Std. dev. Max. Min.	0.81 0.58 0.93 4.72 0.00	31.17 31.79 4.70 39.63 25.56	9.89 8.96 5.17 25.33 1.37	15.01 14.62 5.74 31.76 5.33	5.13 5.60 3.43 10.04 -4.15
4	London, South East, Wales, East of England	Average Median Std. dev. Max. Min.	0.69 0.00 0.86 3.23 0.00	56.63 55.66 4.72 72.17 49.65	7.21 7.89 2.84 13.47 0.81	8.12 7.84 2.79 14.94 1.55	0.89 1.04 2.70 8.93 -5.83
All high streets		Average Median Std. dev. Max. Min.	0.68 0.34 0.86 5.36 0.00	43.61 44.10 9.26 72.17 17.86	9.00 8.27 4.75 30.23 0.81	11.95 10.62 5.82 34.20 1.55	2.98 2.94 3.54 20.19 -8.49

Table 6 displays the 4 different clusters ranked in order of their average rise in high street vacancy following the easing of retail specific lockdown restrictions and their associated qualities to create a typology of resilience.

**Table 6:** British high street resilience qualities. Estimation method: Hierarchical clustering with spatial constraints

Resilience (Highest to lowest)	Location	Description
Average stability with low pre-pandemic vacancy, high essential retail and services %	London, South East, East of England, Wales	- Average number of new stores opening pre-pandemic. - Low vacancy pre-pandemic. - Many convenience or essential retail stores and services, pre-pandemic. - Lowest increase in vacancy following easing of retail lockdown restrictions.
Stable with moderate pre-pandemic vacancy and essential retail and services %	London, Yorkshire and the Humber, West Midlands, East Midlands, North West, South West, Wales East of England, South East	- Few new stores opening pre-pandemic. - Moderate vacancy pre-pandemic. - Moderate number of convenience or essential retail stores and services, pre-pandemic. - Average increase in vacancy following easing of retail lockdown restrictions.
Unstable with high pre-pandemic vacancy, low essential retail and services %	Scotland, North West, North East, East Midlands, Yorkshire and the Humber, West Midlands	- Many new stores opening pre-pandemic. - High vacancy pre-pandemic. - Low proportion of convenience or essential retail stores and services, pre-pandemic. - Moderate increase in vacancy following easing of retail lockdown restrictions.
Unstable with average pre-pandemic vacancy, lowest essential retail and services %	London, South West, South East, East of England, Wales	- Many new stores opening pre-pandemic. - Average vacancy pre-pandemic. - Lowest proportion convenience or essential retail stores and services, pre-pandemic. - Highest increase in vacancy following easing of retail lockdown restrictions.

**Fig. 2:**
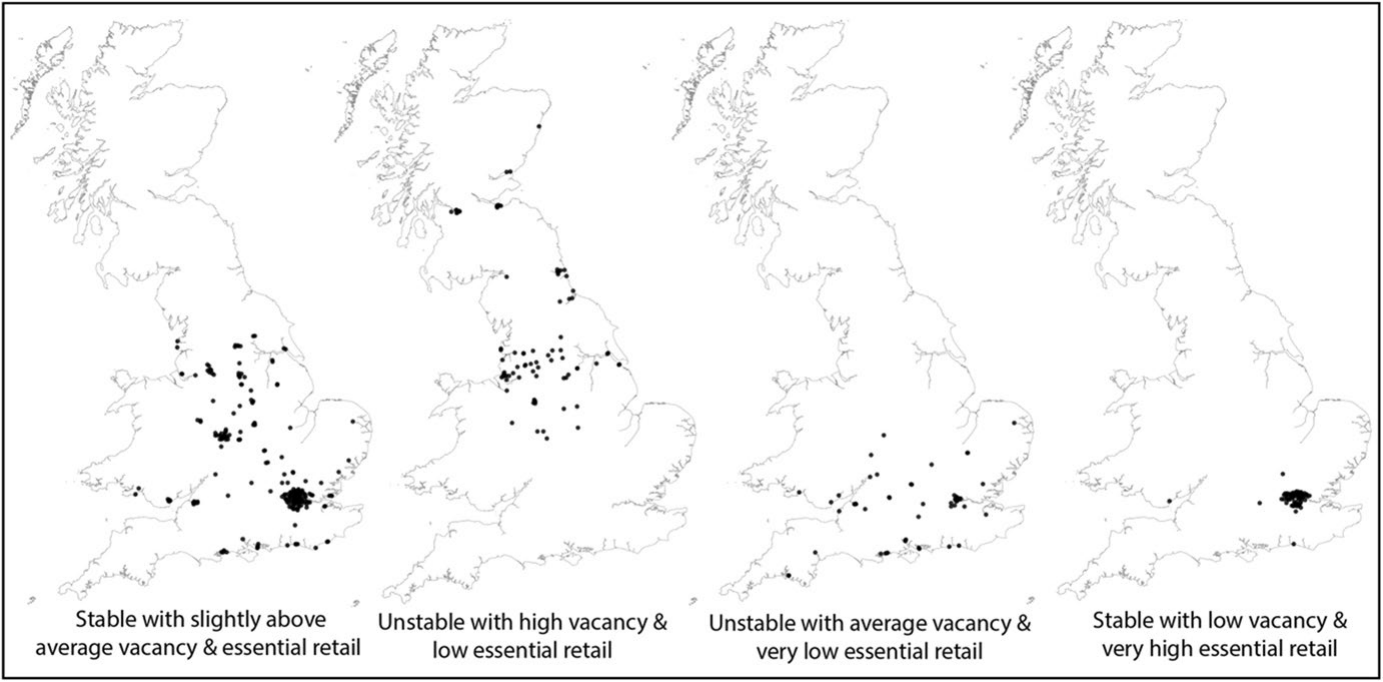
Mapped distributions of high streets based on their cluster allocations (detailed in Table 6)

Figure 2 displays the 4 different clusters visually as the centroid of each high street boundary with their associated cluster and resilience qualities.

The findings from the hierarchical clustering with spatial constraints show that the most resilient high streets to the impact of the retail specific lockdown restrictions were those with average occupier change averaging at 0.69% and the lowest average vacancy of 7.21% in the first 6 months of 2019, before the pandemic hit. The high streets predominantly situated in London, and the East of England, with the additions of Southern Cross, Brighton and Splott, Cardiff, also have the highest proportion of essential retail stores or services at an average of 56.63% of stores allowed to remain open during the retail specific lockdown restrictions. As a result, the most resilient cluster had an average rise in high street vacancy of 0.89%. In contrast, the least resilient cluster of high streets had an average increase in vacancy of 5.13% and are situated within the regions of the South West, South East, London, East of England and Wales. The cluster of high streets with the lowest resilience, also had an unstable retail environment with the proportion of occupier change in January-June of 2019 to be 0.81%. Interestingly, the cluster of high streets had an average vacancy percentage for the first 6 months of 2019 at 9.89%. However, the cluster did have the lowest proportions of essential stores or services which were allowed to remain open at 31.17%.

**Fig. 3:**
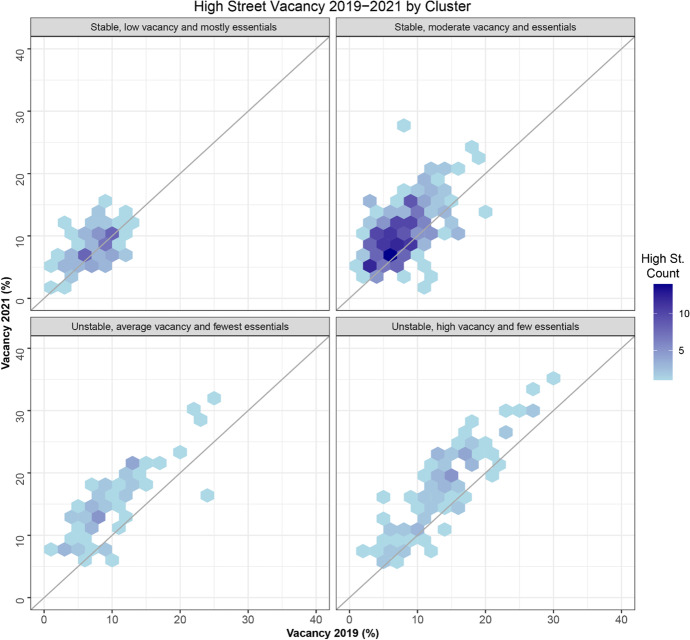
Hexagonal heatmap of 2d bin counts for 2019 vacancy against 2021 vacancy according to the 4 clusters detailed in Table 6

**Fig. 4:**
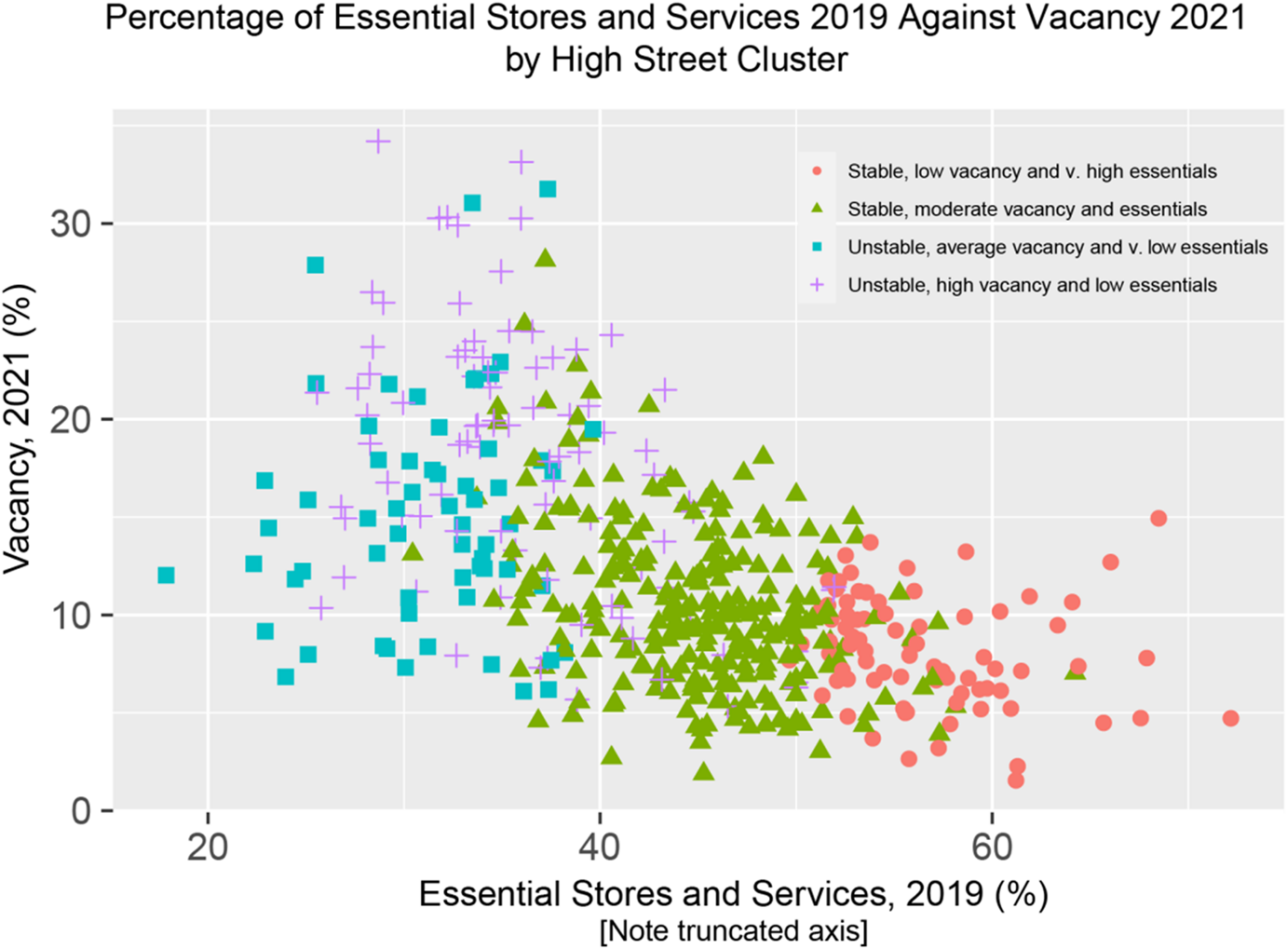
Scatterplot for essential stores and services (2019) and vacancy (2021) coloured by classification detailed in Table 6

Figure 3 displays a hexagonal heatmap of the 2d bin counts for each individual classified hierarchical cluster with spatial constraints for vacancy before the COVID-19 pandemic and after all lockdown restrictions to retail were lifted. Figure 4 shows a scatterplot for the relationship between the proportion of essential stores and services allowed to remain open during the COVID-19 retail specific lockdown restrictions and vacancy % following the easing of those restrictions. The scatterplot displays the relationship per hierarchical cluster and outlines some specific high streets.

## Discussion

High streets with higher proportions of stores deemed by the government as essential before the lockdown restrictions in 2019, had lower rates of vacancy following the lifting of the restrictions. Additionally, high streets that had high proportions of essential services and low vacancy before the pandemic, had resulting lower rates of vacancy in 2021, following the end of retail specific lockdown restrictions. Therefore, when the pandemic hit, pre-existing successful high streets with higher levels of essential stores and services were allowed to keep a higher proportion of their stores open during the lockdown, making them more resilient.

For those high streets with high vacancy before the pandemic, Grenadier’s (1995) work on real estate cycles can be applied to explain the reluctance of landlords to fill vacant spaces in downward markets, as seen during the 2008 recession. This is because it is a more cost-effective option for landlords to retain the current environment of vacancy is enough to offset the possible benefits of filling a vacant space. The pandemic has contributed to the persistence of prolonged cycles in the retail real estate market due to demand uncertainty, adjustment costs, and lags in construction.

The second part of this research developed a typology of high street resilience based on the indicators of vacancy, essential stores or services proportion, occupier change and geographical proximity. It revealed that some high streets in London, the South East, the East of England and Cardiff with average occupier change, low pre-pandemic vacancy and high essential stores or services and convenience proportion to have had the lowest rise in vacancy following the lockdown restrictions. This group of high streets included Northolt Road in Harrow, London, with the highest proportion of essential stores at 72%, and Wanstead High Street, London, which had the lowest vacancy rate following the lockdown restrictions at 2%.

The second most resilient category of high streets had pre-pandemic conditions including very low occupier churn, moderate vacancy, and an ample level of stores allowed to remain open during the lockdown restrictions. Within this category is Stockport Road in Manchester, which only had a 2% rise in vacancy after the COVID-19 restrictions due to approximately half of the stores on the high street being allowed to remain open. These high streets managed to retain their prior success through having pre-exiting stores that sufficiently met the local consumers’ needs and were mostly able to remain open for business during March 2020 to May 2021.

In contrast, high streets in London, the South West, South East, East of England and Wales with characteristics before the pandemic including: the opening of many new stores, average level of vacancy and an extremely low proportion of stores that were allowed to remain open during the lockdown restrictions were found to have the highest average rise in vacancy. Within the least resilient cluster of high streets is Spring Street in Paddington, London, which had a rise of 13% in vacancy. The disproportionate rise in vacancy could arguably lie in the reduced commuter population to the area caused by the lockdown restrictions. These non-resilient high streets also had an above average proportion of new stores opening. However, these new stores were perhaps not in line with the wider cultural changes to consumer behaviour (Coca-Stefaniak, 2013; Dolega & Lord’s, 2020) – specifically, the persistent growth in convenience culture, a trend arguably exacerbated through the lockdown orders to shop locally. The instability of these high streets should be of particular importance to policy makers.

The overall findings from the resilience classification could be interpreted as quantifying Appel and Hardaker’s (2021) in-depth interviews that categorised retailers by their strategies to either return to their prior success or to attain sustainable growth by making fundamental change to adapt to fit in with the relentless encroach of the online realm into our lives. Our study found that those high streets close to the average number of store openings to be more resilient to the immediate impacts of the COVID-19 pandemic and restrictions. Such high streets also exhibited low vacancy rates to suggest that the new stores that were opening fitted in with wider societal and cultural trends making them more resilient to the economic and restrictive implications of the pandemic. One such example is Trafalgar Road in Greenwich, London, which saw multiple new stores occupy previously vacant locations, including cafés and bakeries with outside seating. The same type of high streets also had the highest proportions of essential stores and services before the pandemic. Therefore, it could be suggested that the new stores opening on these resilient high streets were either in line with the rise in convenience culture or were able to adapt to provide ‘pandemic-friendly’ options such as takeaway service or outdoor seating. The lack of restrictions on these new businesses and an additional rise in demand for their products following panic buying and consumption displacement, arguably gave these new stores a greater chance of survival (Hall et al., 2020). While this study has focused on distinctions between high streets based on the number of stores allowed to remain open during the lockdowns, there is the possibility that the regression model omitted essential explanatory variables leading to errors. Such potential explanatory factors include the impact of the 3 and subsequently 4 Tier system in England between 14th October 2020 and 6th January 2020 where local restrictions were enforced. Unevenly distributed restrictions across English authorities may have led to disproportionate economic impact across high streets. Other influential factors might include high street businesses response to Government support including business rates relief, the lease forfeiture moratorium and the furlough scheme.

Identifying biased coefficients and error terms related to omitted variables cannot be achieved through use of this paper’s existing methods. Nevertheless, all the variables selected within the regression model were grounded in pre-existing literature and the findings were in line with previous studies on retail resilience based on spending data and qualitative research.

Through reporting geographical disparities in high street resilience, this paper has aimed to inform discussion related to policy tools for town centre recovery and revitalisation following the lockdowns. Large, up-to-date granular data sets such as that of the Local Data Company provides the possibility for local authorities to understand their high streets’ composition and resilience qualities. In particular, geographically targeted policies can be tailored to provide intervention to areas that could benefit from more stable occupier conditions or to provide support to areas that are already adaptively resilient. 

## Data Availability

The dataset used for this research was released by the Local Data Company.

## Appendix 1

List of the categories of occupier types within the Local Data Company data set on retail premises location. The store categories have been deemed as essential due to their ability to remain open during the retail related lockdown restrictions in England, Wales and Scotland during March 2020 to May 2021.

**Table:**

Essential stores and services		
Accident compensation	Discount store	Laser eye treatment
Accountants	Doctors surgeries	Laundries and launderettes
Advice centres	Dry cleaners	Letting agents
Alternative and complementary medicines	Electricians	Livery companies
ATM lobby	Estate agents	Locksmiths
Bakers shops	Farmers markets	Minicabs
Banks and other Financial institutions	Fast food delivery	Mobility services
Builders	Fast food takeaway	Mortgage companies advisors
Builders merchant	Financial advisors	MOT testing centres
Building societies	Fish and chip shops	Newsagents
Bureaux de change	Fishmongers	Nursing homes
Butchers	Funeral directors and monumental masons	Opticians
Coffee shops	Garage services	Painting and decorating supplies
Café and tearoom	Garden and patio furniture	Pet shops and pet supplies
Community centres	Garden centres	Petrol filling stations
Car and van hire	Gas log and coal fires	Pizza takeaway
Car accessories and parts	Gas suppliers	Plumbers
Car body repairs	Gourmet food	Plumbers merchants
Car breakdown and recovery service	Greengrocers and fruit sellers	Post office services
Car parking and garaging	Grocers	Postal, packing and shipping
Car wash and valet services	Halal butchers	Property consultants
Cash and carry	Halls	Recruitment agencies
Central heating- installation and servicing	Hardware merchants and ironmongers	Royal mail delivery offices
Chartered surveyors	Health centre	Safe deposit boxes
Cheese shops	Health clinics	Shoe repairs
Chemists and toiletries	Health clubs	Solicitors
Cheque cashing	Health foods and products	Storage and removals
Child care	Hearing aids	Street markets
Chinese fast food takeaway	Herbalists	Student unions
Chocolatiers	Hire centres	Supermarkets
Citizen advice bureaux	Household services	Take away food shops
Confectioners	Household stores	Taxis and private hire
Convenience stores	Housing association	Tea and coffee merchants
Cosmetic dentistry	Hydroponics	Translation agencies
Cosmetic surgery	Indian takeaway	Tyre dealers
Council services	Indoor markets	Uniforms and staff wear
Credit unions	Information centre	Universities and colleges
Cycle shops	Insurance agents	Veterinary surgeons and practitioners
D.I.Y	Ironmongers	Wholesalers
Delicatessen	Job centres	Wine making and brewer supplies
Delivery services	Joiners	Wines, spirits and beers
Dentists	Landscape gardeners	
Depot	Language schools	
